# A non-randomised feasibility study of a mHealth follow-up program in bariatric surgery

**DOI:** 10.1186/s40814-023-01401-3

**Published:** 2023-10-17

**Authors:** Charlene Wright, Jaimon T. Kelly, Joshua Byrnes, Katrina L. Campbell, Rebecca Healy, Jane Musial, Kyra Hamilton

**Affiliations:** 1 School of Applied Psychology, Griffith University, 176 Messines Ridge Road, Mount Gravatt, QLD Australia; 2 Menzies Health Institute Queensland, Griffith University, 176 Messines Ridge Road, Mount Gravatt, QLD 4122 Australia; 3 Centre for Online Health, Faculty of Medicine, The University of Queensland, Woolloongabba, QLD Australia; 4 Centre for Health Services Research, Faculty of Medicine, The University of Queensland, Herston, QLD Australia; 5 Centre for Applied Health Economics, School of Medicine and Dentistry, Griffith University, 170 Kessels Road, Nathan, QLD Australia; 6 Healthcare Excellence and Innovation, Metro North Hospital and Health Service, Butterfield St, Herston, QLD Australia; 7 Nutrition and Dietetics Department, Royal Brisbane and Women’s Hospital, Butterfield St, Herston, QLD Australia; 8 Nutrition and Dietetics Department, Princess Alexandra Hospital, 199 Ipswich Road, Woolloongabba, Australia; 9 Health Sciences Research Institute, University of California, 5200 Lake Road, Merced, CA 95343 USA; 10 Faculty of Sport and Health Sciences, University of Jyväskylä, Jyväskylä, Finland

**Keywords:** Bariatric surgery, mHealth, Mixed methods, Text messages, Email newsletter, Online resources, Feasibility, Acceptability, Cost analysis

## Abstract

**Background:**

Behavioural support via mobile health (mHealth) is emerging. This study aimed to assess the feasibility, acceptability, cost, and potential effect on weight of a mHealth follow-up program in bariatric surgery.

**Methods:**

This was a non-randomised feasibility study describing intervention development and proof in the concept of a mHealth follow-up program in bariatric surgery. The study compares a prospective cohort with a historical control group and was conducted in a tertiary bariatric surgery service in Australia. The intervention group included individuals who had bariatric surgery (2019–2021) and owned a smart device, and the historical control group received usual postoperative care (2018). The intervention involved usual care plus codesigned biweekly text messages, monthly email newsletters, and online resources/videos over a 6-month period. The primary outcome measures included feasibility (via recruitment and retention rate), acceptability (via mixed methods), marginal costs, and weight 12 months postoperatively. Quantitative analysis was performed, including descriptive statistics and inferential and regression analysis. Multivariate linear regression and mixed-effects models were undertaken to test the potential intervention effect. Qualitative analysis was performed using inductive content analysis.

**Results:**

The study included 176 participants (*n* = 129 historical control, *n* = 47 intervention group; mean age 56 years). Of the 50 eligible patients, 48 consented to participate (96% recruitment rate). One participant opted out of the mHealth program entirely without disclosing their reason (98% retention rate). The survey response rate was low (*n* = 16/47, 34%). Participants agreed/strongly agreed that text messages supported new behaviours (*n* = 13/15, 87%); however, few agreed/strongly agreed that the messages motivated goal setting and self-monitoring (*n* = 8/15, 53%), dietary change (*n* = 6/15, 40%), or physical activity (*n* = 5/15, 33%). Interviews generated four main themes (*n* = 12): ‘motivators and expectations’, ‘preferences and relevance’, ‘reinforced information”, and ‘wanting social support’. The intervention reinforced information, email newsletters were lengthy/challenging to read, and text messages were favoured, yet tailoring was recommended. The intervention cost AUD 11.04 per person. The mean 12-month weight was 86 ± 16 kg and 90 ± 16 kg (intervention and historical control) with no statistically significant difference. Intervention recipients enrolled at 3 months postoperatively demonstrated a statistically significant difference in 12-month weight (*p* = 0.014).

**Conclusion:**

Although this study observed high rates of recruitment and retention, findings should be considered with caution as mHealth may have been embraced more by the intervention cohort as a result of the 2019 coronavirus pandemic. Of the various digital strategies developed and tested, the text message approach was the most acceptable; however, future intervention iterations could be strengthened through tailoring information when possible. The use of email newsletters and online resources/videos requires further testing of effectiveness to determine their value for continued use in bariatric surgery services.

**Supplementary Information:**

The online version contains supplementary material available at 10.1186/s40814-023-01401-3.

## Key messages regarding the feasibility

- Evidence for mHealth in bariatric surgery is emerging with text messaging approaches as the primary mode of delivery. Assessing the feasibility of a multicomponent intervention with additional digital strategies is therefore warranted.
- Feasibility in this study was assessed using ‘demand’, and the extent to which the mHealth program is likely to be used (i.e. how much demand is likely to exist) was measured via recruitment and retention rate. Although this study observed high rates of recruitment and retention, findings should be considered with caution as mHealth may have been embraced more by the intervention cohort as a result of the 2019 coronavirus pandemic.
- Future iterations of the intervention may prioritise the text messaging approach with tailoring. Email newsletters and online resources/videos require further testing to determine their value in bariatric surgery services.

## Introduction

Research has shown that outcomes of bariatric surgery are associated with preoperative and postoperative psychological and behavioural factors [1–4]. Successful bariatric surgery outcomes can depend on the patient’s understanding of the role of bariatric surgery in weight management and making substantial changes to their eating patterns, adopting regular physical activity, and maintaining these behaviours [5, 6]. The management of human behaviour is complex, and bariatric surgery adds further complexity. There is a recognised need for additional lifestyle management of patients undergoing bariatric surgery [6], and it is recommended that preoperative- and postoperative follow-up of patients involve behaviour modification implemented by a multidisciplinary team [6, 7].

A multidisciplinary team is defined as three or more health professionals committed to a shared purpose with complimentary but individual goals [8]. For example, the surgeon’s role can include establishing and discussing the risks and benefits of bariatric surgery and the different procedural options, performing the surgical procedure, consulting patients postoperatively, evaluating diet progression and tolerance, and investing causes of postoperative complications [6]. The dietitian’s role can include performing dietary assessments and counselling to help patients undergo behaviour changes consistent with surgery [9]. Dietary recommendations for continued weight loss after bariatric surgery include consuming adequate fluid, meeting protein requirements, limiting simple sugars, avoiding eating and drinking simultaneously, eating slowly, chewing food extensively, and taking a daily multivitamin [6].

In addition to dietary recommendations, incorporating regular physical activity is a standard recommendation to assist patients with muscle mass maintenance during weight loss and aid weight maintenance [10]. Patients are also encouraged to participate in self-monitoring and incorporate mobile health (mHealth) using various delivery methods [6]. Self-monitoring can lead to improved weight loss results [11], and the incorporation of mHealth shows promising results regarding additional or alternative low-cost patient support modalities [6].

The feasibility and potential for mHealth and electronic health (eHealth) to effect behaviour change are emerging [12], and in bariatric surgery, it is showing some promising results in improving postoperative weight loss and eating psychopathology assessment measures [6, 13]. mHealth is the use of mobile wireless technologies for public health and includes text messaging programs, mobile applications, and wearable devices [14]. A benefit of mHealth is that it can enable the provision of a ‘package of care’ to offer additional lifestyle support [15]. Although various digital strategies can be delivered via mHealth currently, evidence for the effective use of digital technologies in bariatric surgery is mostly focused on text messaging approaches. Lemanu et al. conducted a randomised controlled trial with bariatric surgery patients and found that a text message intervention improved adherence to exercise advice [16]. Furthermore, Lauti et al. conducted a randomised controlled trial with patients following laparoscopic sleeve gastrectomy and found text message support is feasible, may reduce weight regain, and is valued [17]. While bariatric surgery patients have a preference for text messaging, they also report a desire for email approaches and want additional postoperative support, information, and resources from their bariatric surgery health service [18]. It seems warranted therefore that further evaluation of an intervention using multiple digital strategies is undertaken.

This study will describe the intervention development and, with a mixed-methods approach, provide proof of concept and evidence for a mHealth follow-up program in bariatric surgery. Studies that describe intervention development typically adopt qualitative methods [19, 20]. Integrating quantitative and qualitative data can assist with developing comprehensive and nuanced understandings of feasibility [21]. The intervention delivered in this study was developed following the French et al. model [22]. Steps in the model include the following: (1) identifying the target behaviours, population, and context, (2) assessing the problem, (3) forming possible solutions, and (4) evaluating the intervention [22]. The developed mHealth program utilised text messages, email newsletters, online resources, and videos for postoperative bariatric surgery patients that could be accessed via a smartphone or tablet. This study aimed to assess the mHealth program’s feasibility, acceptability, cost, and potential effect on weight. It was expected that all components of the mHealth program would be feasible and acceptable to patients. Furthermore, it was expected that the mHealth program may have the potential to result in improved weight loss 12 months postoperatively when compared to a historical control group receiving usual care.

## Materials and methods

### Study design, setting, and sample

This non-randomised feasibility study evaluated a mHealth follow-up program in bariatric surgery. The evaluation compared a prospective cohort and historical control group, assessing the feasibility, acceptability, cost, and potential effect on weight. A mixed-methods approach was used, including a survey and semi-structured interviews. Ethical approval was provided by both the hospital recruitment site and collaborating university (LNR/2020/QRBW/63798 and 2020/844). Study findings were reported based on STROBE [23], COREQ [24], and TIDieR guidelines (Tables S1, S2 and S3) [25].

The study was conducted at a tertiary bariatric surgery service in Queensland, Australia. Participants in the intervention group were recruited from July 2020 to July 2021 and received the intervention over a 6-month period. The historical control group included all patients who received usual postoperative care during 2018. The year 2018 was selected as these patients received the usual postoperative care without the potential impacts of the 2019 coronavirus pandemic. Data were retrospectively obtained from medical records over the study period. The sample size was based on practical considerations, including participant flow and budgetary constraints. Furthermore, considering it is an initial evaluation study to provide proof of concept and evidence for the intervention, and did not perform formal hypothesis testing for effectiveness or efficacy, a power analysis was not required [26].

The intervention group eligibility criteria included adults (≥ 18 years) who had undergone bariatric surgery during 2019–2021, were still receiving outpatient postoperative follow-up care in the bariatric surgery service, and owned a smart device capable of receiving text messages or emails (i.e. smartphone or tablet). Potential participants were screened for eligibility by a local site investigator (R. H.) from outpatient appointment lists and relevant hospital databases and invited to participate. Participants could be recruited and commence the intervention following their 3-month, 6-month, or 12-month follow-up appointment. If patients could not be contacted at their outpatient appointment, they were contacted via telephone and invited to participate. The intervention participants provided verbal consent to participate in the study which included the approval of data collection. Data were obtained at regular follow-up appointments. Follow-up data was not available for participants recruited into the intervention at 12-month post-surgery as follow-up appointments beyond this time point are not routine practice. Patients who were 12 months postoperative remained eligible to receive the intervention as it was made available to the entire bariatric surgery service. The historical control group eligibility criteria included adults (≥ 18 years) who had undergone bariatric surgery in 2018. Data were retrospectively obtained from medical records. Consent for the use of medical records was not obtained. The use of this data was deemed low to no risk and approved by the human research ethics committee. Identifiers were removed, and a master file of the data was encrypted and stored on secure storage.

### Intervention development, delivery, and usual care

The intervention content was guided by clinical practice guidelines, which encourage incorporating nutrition changes, performing in at least some physical activity, and participating in self-monitoring and goal setting after bariatric surgery [6]. These postoperative recommendations apply to all postoperative time points, have strong supporting evidence [6], are modifiable at the patient level, and are routinely recommended by health professionals during postoperative appointments. Considering the fundamental behaviour recommendations postoperatively are uniform, the intervention delivered was the same for all intervention participants regardless of their postoperative time point.

**Fig. 1:**
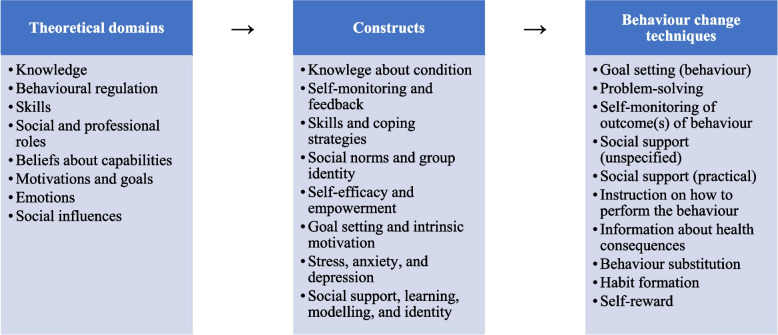
The mHealth program’s theoretical framework guided by the theoretical domains framework and behaviour change technique taxonomy

The mHealth follow-up bariatric surgery program was developed following French et al. [22], and the theoretical domains framework (Fig. 1) was used to inform the pathways of behaviour change [27]. Initially, a mixed-methods approach was adopted to gain further insight from adults undergoing bariatric surgery regarding their barriers to accessing bariatric surgery services, and to explore unmet patient needs, technology use, and digital device preferences, which has been previously published. Knowledge of the barriers to accessing bariatric surgery services further validated the integration of mHealth interventions within the perioperative pathway, and modalities such as text messaging and emails offering additional support, information, and resources regarding diet and physical activity were recommended [18]. In addition, the selection of behaviour change techniques was informed by systematic literature reviews [18, 28] and a matrix that mapped behaviour change techniques to the theoretical domains (Fig. 1) [29].

**Fig. 2:**
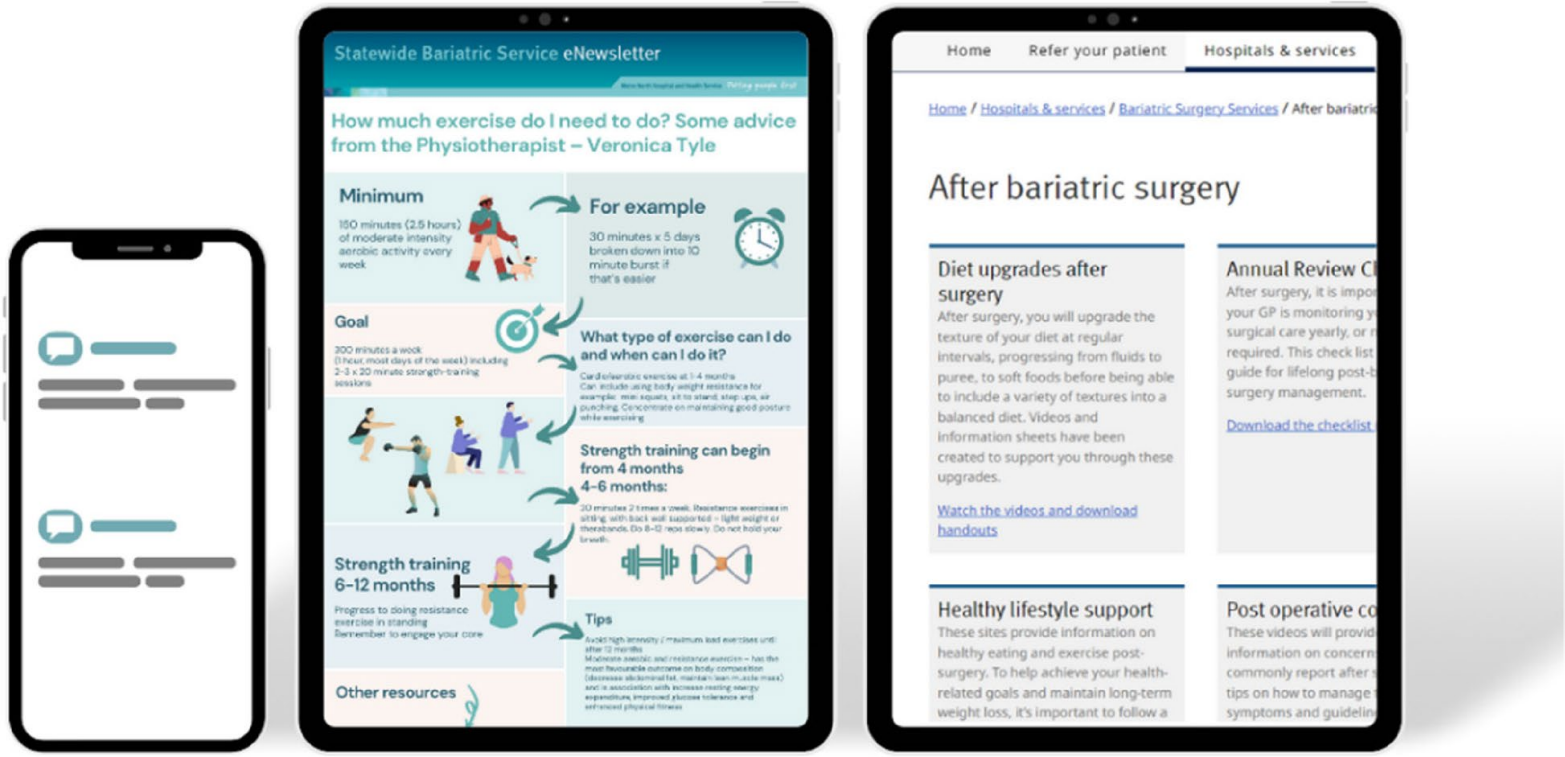
A mHealth program in bariatric surgery delivering text messages, email newsletters, and online resources/videos in addition to usual care

The resultant mHealth follow-up bariatric surgery program was developed to be delivered in addition to usual postoperative care and included text messages, email newsletters, online resources, and videos (Fig. 2, Table S4, and Table S5). The intervention content and delivery frequency were codesigned with clinical members of the research team and a consumer representative who also provided an iterative review of the content.

Text messages were scheduled to be delivered to participants biweekly at 8:00 am, sent from an integrated messaging system. Each text message was mapped to the behaviour change technique taxonomy and the theoretical domains framework (Fig. 1 and Table S4) [27, 30].

The email newsletter was delivered to participants monthly from the hospital’s standard emailing system. A different bariatric surgery multidisciplinary team member curated each newsletter. This included an endocrinologist, dietitian, physiotherapist, psychologist, and surgeon. Topics included ‘protein consumption after surgery’, ‘a reminder from your surgeon after surgery’, ‘medication tips and considerations after bariatric surgery’, ‘introducing exercise following bariatric surgery’, and ‘managing emotional eating’.

Digital resources and videos could be accessed via the organisation’s bariatric surgery services website at the participants’ discretion. Topics included ‘postoperative diet upgrades’, ‘review checklists’, ‘medication changes’, ‘healthy lifestyle support’, ‘postoperative concerns’, and ‘coping tools’ (Table S5). Participants were directed to the website during their follow-up outpatient appointments. In addition, as part of the biweekly text message service, the following message was delivered: ‘How are you tracking with your diet and lifestyle? Remember to check out the Statewide Bariatric Service Website for further information and support’.

Participants could opt out of receiving text messages, email newsletters, or both text messages and email newsletters before study commencement. In addition, they were provided instructions on how to stop them and opt out throughout the study period. The mHealth program was delivered in addition to usual care; however, as of April 2020, telehealth consultations replaced most face-to-face appointments during the study recruitment period due to the 2019 coronavirus pandemic.

Usual care within the tertiary bariatric surgery service in Queensland, Australia, consists of follow-up with a surgeon at 2 weeks postoperatively and follow-up with a dietitian, endocrinologist, and physiotherapist at 1 month, 3 months, 6 months, and 12 months postoperatively. In addition, patients are seen by a psychologist at 6 months postoperatively and other times if identified as high risk. The historical control group received the same usual care as the intervention group with follow-up appointments at 1 month, 3 months, 6 months, and 12 months via face-to-face appointments. Comparatively, the intervention group had most of their appointments via telehealth, and they received the mHealth program.

### Data collection

#### Participant characteristics and feasibility

Demographic data (age, sex, employment status, and Aboriginal and Torres Strait Islander status) and index procedure data (date of surgery, type of surgery, length of hospital stay, and preoperative height, weight, body mass index, and excess weight) were obtained from medical records. The key area of focus for feasibility was ‘demand’ and the extent to which the mHealth program is likely to be used (i.e. how much demand is likely to exist) [31]. This was measured with recruitment and retention rate data, with successful retention and recruitment set at 80%. Those lost to follow-up were not included in the analysis.

#### Usability and acceptability (mixed methods: quantitative survey and qualitative interviews)

Surveys were disseminated to participants via REDCap [32], at least 3 months after intervention commencement. The survey was modelled on a previous study [33] and included 19 items to establish usability and acceptability. Eight questions asked participants to rate the mHealth program on a 5-point Likert scale, seven were yes/no questions, two were multiple-choice questions, and two were open ended (Table S6).

The interview guide was comprised of open-ended questions (Table S7) and explored the usability and acceptability of the program delivery methods in general, with further questions specific to each of the various digital strategies (text messages, email newsletters, online resources, and videos) and the program content. In addition, it explored behaviour change, goal setting, self-monitoring, and maintaining behaviours and strategies beyond the intervention (Table S7.) For the semi-structured interviews, participants were recruited based on consecutive sampling [34]. On completing the intervention, participants were identified, telephoned, and invited to participate in a semi-structured interview (R. H.) (response rate was 100%). The recruitment continued until no other themes were generated from the data [35]. Interviews were conducted by the lead author (C. W.), a female PhD candidate and accredited practising dietitian, with masters-level qualifications and postgraduate studies relating to bariatric surgery. No relationship was established with participants prior to study commencement, and participants did not know personal details about the researcher. The interviewer has previously conducted mixed-methods and qualitative research with adults undergoing bariatric surgery. The first three interviews conducted served as a pilot test of the interview guide (Table S7); these interviews were included in the final analysis. Two research team members (C. W., J. K.) reviewed the interview guide after the pilot interviews to ensure relevant responses were drawn. After this, four additional questions were added to the interview guide (Table S7). Interviews were scheduled for 30 minutes and occurred from April to July 2021. They were conducted via telephone in a location separate from the recruiting hospital, with no other researchers present. Interviews were audio-recorded on two electronic devices to collect the data and mitigate the risk of data loss. Handwritten field notes were taken and analysed, and the interviewer kept a reflective journal recording ideas and issues expressed, along with the similarities and differences among interviews and possible refinement of questions [36].

#### Cost analysis

A local site investigator (R. H.) obtained the cost data for the mHealth program. The cost of developing the resources and materials was estimated; however, they were considered a sunk cost (research) and not included in the per-person estimate. The cost of establishing the process to enable the mHealth program was considered. The cost associated with delivering the program was calculated, including the cost of administering the email and text message delivery and the service fee of the integrated messaging system.

#### Potential effect on weight

Data on weight (kg) was available for both the historical control group and the intervention group at the time of surgery, 3 months, and 6 months. Weight at 12-month post-surgery was available for the historical control group and participants recruited into the intervention at 3 months and 6 months postoperatively. Weight at 12-month post-surgery was used as the primary outcome for comparison.

### Data analysis

#### Participant’s characteristics

Demographic data were analysed using Stata (version 14) [37]. Continuous variables not normally distributed (length of stay) were reported using median (range). Those normally distributed (age and pre-op anthropometry including height, weight, body mass index, and excess weight) were reported using mean ± standard deviation. All categorical variables (type of surgery, employment status, and Aboriginal and Torres Strait Islander status) were reported as frequency (n) and percentage. Bivariate analysis was conducted to determine similarities in the baseline demographics between the groups, including the chi-square test for categorical variables, independent samples *T*-test for normally distributed continuous variables, or Mann–Whitney *U*-test for those not normally distributed. Significance was determined at the 0.05 significance level.

#### Usability and acceptability (mixed methods: quantitative survey and qualitative interviews)

All survey variables were categorical and reported as frequency (n) and percentage. All percentages refer to the valid data that was available for the variable. Variables without a response were treated as missing data and not analysed.

The lead author conducted an inductive content analysis of the semi-structured interview transcripts in NVivo (version 12) [38, 39]. The analysis process described by Elo and Kyngäs was used as a guide, represented by three main phases: preparation, organising, and reporting [40]. The preparation stage involved becoming immersed in the data and selecting the unit of analysis (usability and acceptability). The organising stage involved open coding and creating categories, grouping codes under higher-order headings, and formulating a general description of the research topic. Themes and subthemes were generated. Next, two research team members reviewed all codes, themes, and subthemes (C. W., J. K.), and any inconsistencies in the coding assignment were resolved through discussion. Finally, the reporting stage included reporting these themes and subthemes, and representative quotes were selected and agreed upon (C. W., J. K.).

#### Cost analysis

A health system perspective was taken when establishing the cost of the mHealth program, and costs borne by the health system were included. The primary outcome was the cost per person. The cost considers the marginal costs associated with delivering the program and is reported in 2020 Australian dollars (AUD). Marginal costs were calculated by the time and cost of employed staff divided by the total number of intervention participants and the service fee charged by a commercial company. The time horizon is limited to the observation period; therefore, the costs associated with future revising or updating of content were not considered, and discounting of future costs was not required.

#### Potential effect on weight

Independent samples *t*-test was conducted to determine if weight (kg) at 12 months postoperatively differed between the intervention and historical control groups. In addition, a multivariate linear regression (controlling for weight at the time of surgery) was conducted to determine if weight at 12 months postoperatively differed between participants recruited into the intervention at 3 months, 6 months, and 12 months and the historical control group. A linear mixed-effects model was conducted to determine if the mean weight differed statistically between the historical control group and participants recruited into the intervention at 3 months and 6 months at different follow-up time points. An interaction term between time and study group (historical control, enrolled into intervention at either 3 months, 6 months, or 12 months) was included to test the difference in weight over each time interval between groups. Weight at the time of surgery was included as a fixed and random effect determined based on a likelihood-ratio test of model fit. Results include expressions of uncertainty with 95% confidence intervals reported.

## Results

### Participant characteristics and feasibility

Fifty patients met the inclusion criteria within the bariatric surgery service and were invited to participate and receive the mHealth program. Of the 50 eligible patients, 48 consented to participate (96% recruitment rate). Participants were recruited at various postoperative time points including 3 months (*n* = 16, 33%), 6 months (*n* = 9, 19%), and 12 months postoperatively (*n* = 23, 48%). One participant opted out of the mHealth program entirely without disclosing their reason (98% retention rate). Three participants opted to receive text messages only, and one opted to receive email newsletters only. The remaining 44 participants opted to receive both modalities. Participants were predominantly female (*n* = 31, 66%), underwent Roux-en-Y gastric bypass (*n* = 42, 89%), and had a mean age of 55 ± 10 years (Table 1). One-hundred and twenty-nine patients formed the historical control group. Baseline demographic characteristics for recipients of the mHealth program (intervention group) and usual care (historical control group) can be seen in Table 1.

**Table 1:** Baseline demographic characteristics for recipients of the mHealth program (intervention group) and usual care (historical control group) (*N* = 176)

Characteristic	Intervention group (*n* = 47) Frequency (%)	Historical control (*n* = 129) Frequency (%)
**Sex, female**	31 (66.0%)	73 (56.6%)
**Age (years)**^**a**^	54.5 ± 9.6	56.1 ± 8.1
**Type of surgery**		
Roux-en-Y gastric bypass	42 (89.4%)	104 (80.6%)
Laparoscopic sleeve gastrectomy	4 (8.5%)	24 (18.6%)
Mini bypass	1 (2.1%)	0 (0.0%)
Single loop	0 (0.0%)	1 (0.8%)
**Preoperative anthropometry**		
Height (m)^a^	1.7 ± 0.1	1.7 ± 0.1
Weight (kg)^a^	118.9 ± 16.4	118.1 ± 17.2
Body mass index (kg/m^2^)^a^	42.0 ± 6.0	42.0 ± 6.5
Excess weight (kg)^a^	55.5 ± 12.7	58.5 ± 18.4
**Employment status**		
Full time	20 (42.6%)	38 (29.5%)
Part time	10 (21.3%)	15 (11.6%)
Home duties/retired	17 (36.2%)	48 (37.2%)
Unemployed	0 (0.0%)	28 (21.7%)
**Aboriginal and Torres Strait Islander**		
Does not identify	31 (66.0%)	103 (79.8%)
Aboriginal	8 (17.0%)	18 (14.0%)
Torres Strait Islander	2 (4.3%)	2 (1.6%)
Aboriginal and Torres Strait Islander	3 (6.4%)	2 (1.6%)
Did not wish to disclose	3 (6.4%)	4 (3.1%)
**Length of hospital stay (days)**^**b**^	2 (1–7)	2 (1–18)

^a^Mean ± standard deviation

^b^Median (range)

Twelve intervention recipients completed a semi-structured interview, of which 58% were male (*n* = 7), with a mean age of 56.8 ± 7.1 years. The mean duration of the interviews was 13 ± 3 minutes (range 10–18 minutes). The majority were recruited into the intervention at their 12-month postoperative appointment (*n* = 7, 58.3%), followed by those recruited 3 months postoperatively (*n* = 3, 16.7%) and 6 months postoperatively (*n* = 2, 25.0%).

### Usability and acceptability (quantitative survey)

**Fig. 3:**
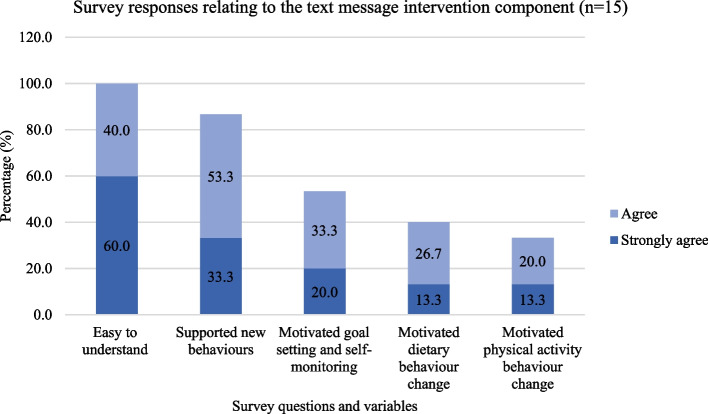
Survey responses specific to receiving the text message component of the mHealth program in bariatric surgery (*n* = 15)

The survey was delivered to the 47 intervention recipients, with a 34% response rate (*n* = 16). The survey results are in Fig. 3, and the complete results are in Table S6. Of the text message recipients, 93% reported always reading them (*n* = 14/15), and 93% approved of the biweekly frequency (*n* = 14/15).

The email newsletter recipients approved of the monthly frequency (*n* = 7/8, 88%), agreed the information provided was useful (*n* = 7/8, 88%), agreed they would always read them (*n* = 6/8, 75%), and agreed/strongly agreed they were easy to understand (*n* = 7/8, 88%).

Over half of the survey respondents did not access the online resources and videos (*n* = 10/16, 63%). The majority of those who accessed the online resources and videos found the information useful (*n* = 5/6, 80%) and easy to understand (*n* = 5/6, 80%). Two participants responded to the open-ended question, which asked how the mHealth program could be improved. They did not comment on the mHealth program but on the positives of having undergone bariatric surgery.

### Usability and acceptability (qualitative interviews)

**Fig. 4:**
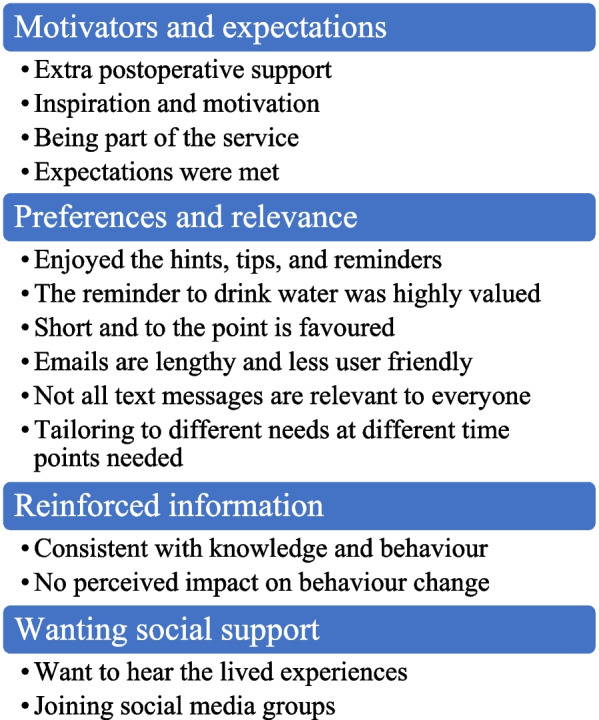
Themes and subthemes generated from the qualitative content analysis of semi-structured interviews with recipients of the mHealth program (*n* = 12)

The interviews generated four main themes: ‘motivators and expectations’, ‘preferences and relevance’, ‘reinforced information’, and ‘wanting social support’. In addition, each of the main themes had several subthemes (Fig. 4). Little variation in responses across the three patient groups was identified; thus, common themes across the groups were reported.

#### Motivators and expectations

Motivators for participating in the study and receiving the mHealth intervention varied. Motivators included wanting extra postoperative support: ‘Any extra information is good information, and it reinforces the information I’ve been given’ (male, 66, 3 months postoperative), as well as inspiration and motivation: ‘An inspirational type thing, motivation, and things like that’ (female, 53, 12 months postoperative). However, for some participants, the fact that the mHealth program was part of the service and free was their motivator ‘To give my support, [and] the fact that I didn’t pay for it’ (male, 54, 12 months postoperative). Most participants believed that the intervention either met their expectations ‘It has been motivating’ (female, 51, 3 months postoperative) or if they did not have any prior expectations; they were happy with the intervention content received: ‘I didn’t expect anything, but now they’re coming through like they’re good’ (male, 53, 3 months postoperative).

#### Preferences and relevance

The email newsletters were said to be lengthy: ‘They’re [emails] lengthy to read’ (male, 63, 12 months postoperative), and reading them on a smart device was challenging: ‘It’s just hard on your phone [reading emails] … It’s very time consuming you have to like make it bigger and smaller and move it; it’s just too hard on a phone’ (female, 51, 3 months postoperative). For most, text messages were preferred to email newsletters as they were short and to the point: ‘I like that [the text messages] were short and to the point … and give you the message you needed’ (female, 45, 6 months postoperative). It was easier to remember the points made in the text messages compared to emails: ‘I don’t remember long things. I only remember short things and that’s why text messages are more beneficial for me’ (female, 53, 12 months postoperative), and text messages were thought to be suitable for a broad population: ‘No matter really who you are you can use it [a text message]’ (female, 53, 12 months postoperative).

Participants enjoyed receiving the hints, tips, and reminders via text messages: ‘The text messages have been good. Got some good hints and tips and ideas’ (female, 53, 12 months postoperative) and helped keep individuals ‘on track’: ‘[You can get] off track a bit, you get a text message, and you go “oh okay righto back on the wagon”’ (female, 65, 12 months postoperative). The specific reminder to consume adequate fluids was valued: ‘The reminders to drink water … all the reminders to help you’ (female, 51, 3 months postoperative). However, participants highlighted that the text message content might only be relevant to some: ‘Some of the [text messages] I found … I could put into practice and some others … I probably just brush past them … not everything sort of suits me’ (male, 63, 12 months postoperative), and tailoring content to different postoperative time points or dietary texture progressions may be beneficial: ‘It would’ve been also more beneficial straight after the surgery, so they had set ones straight after the surgery like you know the first two weeks you are only allowed to have clear liquids. Give me suggestions you know on what kind of liquids’ (male, 53, 3 months postoperative).

#### Reinforced information

The information provided in the mHealth program was said to be consistent with pre-established knowledge: ‘I just viewed it as …reinforced information’ (male, 66, 3 months postoperative) and behaviours: ‘It was already stuff that I was already doing’ (female, 60, 12 months postoperative). Some participants believed that the intervention did not influence behaviour change. It was suggested that the intervention might have more impact on behaviours when unproductive habits return or emerge: ‘I was pretty much doing it all anyway, it’s still fresh. I think give it a bit more time maybe fall into bad habits and routines and that, I think give it more time and they would be more effective as a reminder’ (male, 53, 3 months postoperative).

#### Wanting social support

Throughout the interviews, participants highlighted that more social support imbedded in the intervention would be desirable. Participants felt that hearing about the lived experiences of others would be beneficial: ‘I thought [the mHealth program] would have been … more personalised like about people’s experiences’ (male, 54, 12 months postoperative). To obtain social support, many reported resorting to social media: ‘People get on there [Facebook groups] talking, ask questions … [and users] give people feedback’ (male, 54, 12 months postoperative). One participant highlighted that the information received via the intervention text messages was consistent with that of social media groups: ‘[Content] coming through on the text messages are sort of like “oh yeah that’s interesting [because] I just read that on the Facebook” I'll be reading Facebook and “oh that’s what I got the other day on the text message” … it sort of reinforces it’ (male, 63, 12 months postoperative).

### Cost analysis

The costs of developing the resources and materials were considered a sunk research cost, although it is estimated to be AUD 1919.02 based on the monetarised cost of in-kind support provided, namely the time of employed clinical, information technology, and research staff involved in development. The cost of establishing the process to enable email and text message delivery was considered nil because processes were already established and in use for other reasons within the bariatric surgery service. The cost of administering the email and text message delivery was estimated to be AUD 406.22 (AUD 8.64 per person over the 6-month period). Based on 2.5 hours at AUD 47.79 to administer the email newsletters and 6 hours at AUD 47.79 for text message delivery, divided by the total number of intervention participants (*n* = 47). The service fee of the integrated messaging system, charged at AUD 0.05 per text message by a commercial company, was AUD 2.40 per person over the 6-month period based on delivering two text messages per person per week. Therefore, the total cost was AUD 11.04 per person over the 6-month period.

### Potential effect on weight

The mean ± standard deviation weight was 89.9 ± 15.6 kg in the historical control group and 85.5 ± 15.5 kg in the intervention group at 12 months postoperatively, with no statistically significant difference between the two groups (mean difference = 4.4 kg; [95% *CI*: − 3.9, 12.7 kg], *p* = 0.293).

Those enrolled in the intervention at their 3-month postoperative appointment compared to the historical control group demonstrated a statistically significant difference in 12-month postoperative weight based on multivariate linear regression after controlling for weight at the time of surgery (adjusted mean difference = -10.5kg [95% *CI*: − 18.9, − 2.2 kg], *p* = 0.014). Those enrolled in the intervention at their 6-month postoperative appointment compared to the historical control group did not display a statistically significant difference in 12-month postoperative weight (adjusted mean difference = 2.3 kg; [95% *CI*: − 5.2, 9.8 kg], *p* = 0.543), neither did those who enrolled at their 12-month postoperative appointment (adjusted mean difference = 1.7 kg; [95% *CI*: − 3.2, 6.7 kg], *p* = 0.488).

**Fig. 5:**
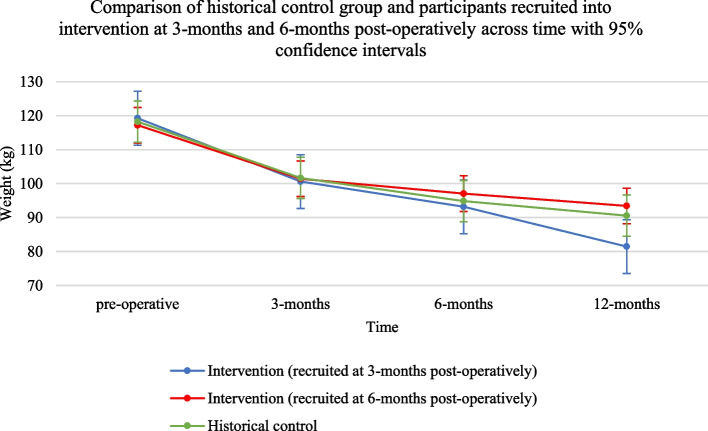
Comparison of the historical control group (usual care) (*n* = 129) and participants recruited into the intervention group (mHealth program) at 3 months postoperatively (*n* = 16) and 6 months postoperatively (*n* = 9) across time with 95% confidence intervals

Results remain consistent when participants recruited at 12 months are excluded from the analysis. Those enrolled in the intervention at their 3-month postoperative appointment compared to the historical control group demonstrated non-statistically significant differences in weight at 3-month and 6-month follow-up but a statistically significant difference in 12-month postoperative weight based on multilevel mixed-effects linear regression (controlling for baseline weight at the time of surgery as a fixed and random effect). The statistically significant adjusted mean difference between the 6-month and 12-month follow-up is -10.1 kg (95% *CI*: − 15.5, − 4.7 kg, *p* < 0.001) (Fig. 5).

## Discussion

This study aimed to provide proof of concept and evidence for a mHealth program, developed as an adjunct to usual care in a tertiary bariatric surgery service, by examining the program’s feasibility, acceptability, cost, and potential effect on weight. The developed mHealth program may be feasible as both recruitment and retention rates were high. The text message approach was found to be acceptable and suitable for continued use in practice. There was no difference in weight between groups; however, intervention recipients enrolled at 3 months postoperatively demonstrated a statistically significant difference in 12-month weight.

The text message component of the intervention, providing frequent reminders and content, was favoured by participants as they were short and to the point. However, participants emphasised that the content might only be relevant to some, and that tailoring may be required. Therefore, future iterations of the intervention could be strengthened by tailoring the content to ensure that the messages are relevant to individual needs. Tailored interventions have more efficacy over time than interventions that base their tailoring on single or infrequent assessments [41]. It could be beneficial to tailor information as patients progress through different postoperative dietary texture progressions or change their behaviours. However, it is expected that such dynamic systems may be more costly to maintain. Suppose a health service solely had access to the automated delivery of scheduled content, considering the text messages were low cost at AUD 0.05 per message and were acceptable by participants, this approach would be suitable for continued use. The recognised need to tailor the intervention does, however, support future research into the development and evaluation of adaptive interventions. Just-in-time adaptive interventions, for example, provide adaptive support (i.e. the type, timing, and intensity) in the moment/context that the person needs it most and is most likely to be receptive [42]. In addition, in different contexts, such as the private health system, where the level of bariatric surgery follow-up care can be highly variable across medical institutions, alternate modalities, such as mobile applications, may be worth further investigation.

The email newsletters were considered lengthy, and reading them on a smart device was challenging. This finding is intriguing as prior formative research indicated that patients prefer to receive or access additional health information via email (*n* = 84, 82%) [18]. This may be explained by the principles of mobile microlearning [43], as it is more challenging to divide content into small, focused units via an email approach. Future iterations of the intervention could be strengthened by optimising email newsletters for mobiles. Currently, despite patients’ preference for email [18], the appropriateness of using email newsletters in routine practice requires further testing. While there was limited uptake of the online resources and videos, individuals undergoing bariatric surgery are known to seek information online as it is readily available and helps them feel informed [18]. A strategy to strengthen future iterations of the intervention could be for the multidisciplinary team to direct patients to the online resources more frequently. Alternatively, the multidisciplinary team could direct patients to pre-existing external online resources that are considered credible and discuss these regularly.

Participants believed that the mHealth program reinforced information provided in usual care and was consistent with existing knowledge and behaviours. The text message component was thought to have minimal impact on participants’ motivation to engage in goal setting and self-monitoring or initiate dietary or physical activity behaviour change. It could be speculated that the mHealth program may have facilitated behaviour change maintenance rather than initiating behaviour change. Further research quantitatively assessing the effect of mHealth on behaviour change or behaviour change maintenance in bariatric surgery is required.

Participants believed that a component missing from the mHealth program was the provision of experiential advice and reported turning to online health communities for social support. This finding is consistent with prior research [18], and bariatric surgery health services may consider facilitating or recommending specific social support groups. Social support is associated with reduced weight regain [2]; however, health professionals and researchers have concerns with the credibility of information shared via online health communities [44]. Further research examining the role of online support groups in facilitating social support for individuals undergoing bariatric surgery is recommended, particularly focusing on distinguishing user-level trust in the information received online and user perspectives regarding the reasons for engagement with online support groups.

Compared to the historical control group, those enrolled in the intervention at their 3-month postoperative appointment demonstrated a statistically significant difference in 12-month postoperative weight. This statistically significant difference appeared between their 6-month and 12-month follow-up appointments. Currently, there is yet to be a consensus on when to commence behavioural interventions in addition to bariatric surgery. The findings from this study indicate that there may be a benefit in introducing the intervention from as early as 3 months postoperatively. However, most weight loss occurs within the first 12 months postoperatively, and patients can experience weight regain and return of comorbid conditions in the medium to long term [45, 46]. Furthermore, patients experience ongoing psychosocial challenges postoperatively, including the fear of weight regain, body image concerns, and navigating negative confrontations and perceived stigma [47]. To provide long-term psychological and lifestyle support and reduce the potential for weight regain, a follow-up care package that commences closer to when patients are susceptible to weight regain and these challenges may be beneficial. Further research about the timing of postoperative support programs in bariatric surgery services would be beneficial.

### Limitations

This study has limitations worth noting. First, the sample size and available data for the quantitative survey were small; therefore, caution should be taken when interpreting results. For the qualitative interviews, recruitment continued until no other themes were generated from the data, indicating a sufficient sample size [35]. Notably, however, there was no qualitative data for the historical cohort which would have allowed for further comparison with the intervention group. Second, results only pertain to those who responded to the survey, participated in the interview, or had complete data available. Thus, insight may be limited to those likely to be engaged with the intervention or bariatric surgery service. These individuals may have more favourable postoperative outcomes or positive experiences than those less likely to attend follow-up appointments. Also, they may have fewer support needs or behaviour change challenges than those lost to follow-up with the bariatric surgery service. Furthermore, a quantitative assessment of the intentions toward or participation in dietary and physical activity behaviours is recommended.

Related to this, there were no effectiveness data other than the potential effect on weight, reflecting the pragmatic nature of the study and data collection in practice. Cost-effectiveness via quality-adjusted life-years was not determined, which is an outcome measure that combines both the duration and quality of life [48], and allows decision-makers to choose between treatment options based on how much they prolong the lives and improve the health of patients. While we demonstrated that the intervention was not expensive, the economic evaluation was limited. The average cost of delivering the program was assumed to be equal to the marginal cost; however, the expected cost per person could increase or decrease depending on high or low numbers of program participants due to variabilities in time associated with the intervention delivery. We recommend that future studies include various patient-reported outcome measures including quality of life, anxiety and depression, and cognitive and behavioural components of eating and economic evaluation.

Notably, intervention participants recruited at the various time points would be at different stages in their recovery; therefore, they may benefit differently from the intervention. Future research should investigate the intervention effect of when participants enter interventions postoperatively. Finally, the study utilised a historical control group where the data was as similar as possible to patients enrolled in the intervention. Thus, the historical control group was considered to be compatible with the intervention group. However, beyond the standard baseline characteristics, an important consideration is that the intervention group was subject to the 2019 coronavirus pandemic, whereas the historical control group was not. Because of the pandemic, and the shift toward telecommunications during this period, mHealth may have been embraced more by the intervention cohort. This could have skewed the recruitment and retention rate which were both found to be high. Caution should be taken when interpreting the recruitment and retention rate. Furthermore, the follow-up care provided in usual care may have changed between the two time points. For example, this could be due to staff changes, staff professional development, or evolving clinical practice standards. These factors could result in the groups being unmatched and potentially less comparable.

## Conclusion

Although this study observed high rates of recruitment and retention, findings should be considered with caution as mHealth may have been embraced more by the intervention cohort as a result of the 2019 coronavirus pandemic. Of the various digital strategies, developed and tested, the text message approach was the most acceptable; however, future intervention iterations could be strengthened through tailoring information when possible. The use of email newsletters and online resources/videos requires further testing of the effectiveness to determine their value for continued use in bariatric surgery services.

### Supplementary Information

**Additional file 1: Table S1.** COREQ Guideline - a 32-item checklist for qualitative studies. **Table S2.** STROBE Guideline - checklist of items that should be included in reports of observational studies. **Table S3.** TIDieR Guideline – checklist of information toinclude when describing an intervention. **Table S4.** Text messages mapped to behaviour change techniques as per the behaviour change technique taxonomy and mechanism of action. **Table S5.** Outline of the digital online resources and videos. **Table S6.** Survey assessing the acceptability and usability of the mHealth program (*N*=16). **Table S7.** Semi-structured interview guide. **Table S8.** Baseline demographics between participants recruited into the intervention at 12-months, 3-months, or 6-months.

## Data Availability

The datasets used and/or analysed during the current study are available from the corresponding author on reasonable request.

## Abbreviations

AUD: Australian dollars
COREQ: Consolidated criteria for reporting qualitative research
eHealth: Electronic health
kg: Kilograms
mHealth: Mobile health
STROBE: The Strengthening the Reporting of Observational Studies in Epidemiology
TIDieR: Template for Intervention Description and Replication
