# Supplementary material for: A non-randomised feasibility study of a mHealth follow-up program in bariatric surgery
Source: Pilot Feasibility Stud. 2023 Oct 17;9:176. doi: 10.1186/s40814-023-01401-3 (PMC10580544; doi:10.1186/s40814-023-01401-3)
Supplement: Supplementary file 1 — Additional file 1: Table S1. COREQ Guideline - a 32-item checklist for qualitative studies. Table S2. STROBE Guideline - checklist of items that should be included in reports of observational studies. Table S3. TIDieR Guideline – checklist of information toinclude when describing an intervention. Table S4. Text messages mapped to behaviour change techniques as per the behaviour change technique taxonomy and mechanism of action. Table S5. Outline of the digital online resources and videos. Table S6. Survey assessing the acceptability and usability of the mHealth program (N=16). Table S7. Semi-structured interview guide. Table S8. Baseline demographics between participants recruited into the intervention at 12-months, 3-months, or 6-months. [file 40814_2023_1401_MOESM1_ESM.docx]

**Supplementary File**

**Table S1:** COREQ Guideline - a 32-item checklist for qualitative studies

**Table S2:** STROBE Guideline - checklist of items that should be included in reports of observational studies

**Table S3:** TIDieR Guideline – checklist of information to include when describing an intervention

**Table S4:** Text messages mapped to behaviour change techniques as per the behaviour change technique taxonomy and mechanism of action

**Table S5:** Outline of the digital online resources and videos

**Table S6:** Survey assessing the acceptability and usability of the mHealth program (N=16)

**Table S7**: Semi-structured interview guide

**Table S8**: Baseline demographics between participants recruited into the intervention at 12-months, 3-months, or 6-months

**Table S1:** COREQ Guideline - a 32-item checklist for qualitative studies

|  | No. Item | Guide Questions/Description | Page # |
| --- | --- | --- | --- |
| Domain 1: Research Team and Reﬂexivity | | | |
| Personal Characteristics | | | |
| Interviewer/facilitator | 1 | Which author/s conducted the interview or focus group? | 12 |
| Credentials | 2 | What were the researcher’s credentials? e.g., PhD, MD | 12 |
| Occupation | 3 | What was their occupation at the time of the study? | 12 |
| Gender | 4 | Was the researcher male or female? | 12 |
| Experience and Training | 5 | What experience or training did the researcher have? | 12 |
| Relationship With Participants | | | |
| Relationship Established | 6 | Was a relationship established prior to study commencement? | 12 |
| Participant Knowledge of The Interviewer | 7 | What did the participants know about the researcher? e.g., personal goals, reasons for doing the research | 12 |
| Interviewer Characteristics | 8 | What characteristics were reported about the interviewer/facilitator? e.g., Bias, assumptions, reasons, and interests in the research topic | 12 |
| Domain 2: Study Design | | | |
| Theoretical Framework | | | |
| Methodological Orientation and Theory | 9 | What methodological orientation was stated to underpin the study? e.g., grounded theory, discourse analysis, ethnography, phenomenology, content analysis | 13 |
| Participant Selection | | | |
| Sampling | 10 | How were participants selected? e.g., purposive, convenience, consecutive, snowball | 11 |
| Method of Approach | 11 | How were participants approached? e.g., face-to-face, telephone, mail, email | 11-12 |
| Sample Size | 12 | How many participants were in the study? | 16 |
| Non-Participation | 13 | How many people refused to participate or dropped out? Reasons? | 11-12 |
| Setting | | | |
| Setting of Data Collection | 14 | Where was the data collected? e.g., home, clinic, workplace | 12 |
| Presence of Non-Participants | 15 | Was anyone else present besides the participants and researchers? | 12 |
| Description of Sample | 16 | What are the important characteristics of the sample? e.g., demographic data, date | 15-16 |
| Data Collection | | | |
| Interview Guide | 17 | Were questions, prompts, guides provided by the authors? Was it pilot tested? | Table S6 & page 12 |
| Repeat Interviews | 18 | Were repeat interviews carried out? If yes, how many? | No |
| Audio/Visual Recording | 19 | Did the research use audio or visual recording to collect the data? | 12 |
| Field Notes | 20 | Were ﬁeld notes made during and/or after the interview or focus group? | 12 |
| Duration | 21 | What was the duration of the interviews or focus group? | 12 |
| Data Saturation | 22 | Was data saturation discussed? | 12 & 24 |
| Transcripts Returned | 23 | Were transcripts returned to participants for comment and/or correction? | No |
| Domain 3: Analysis And ﬁndings | | | |
| Data Analysis | | | |
| Number of Data Coders | 24 | How many data coders coded the data? | 13 |
| Description of the Coding Tree | 25 | Did authors provide a description of the coding tree? | NA |
| Derivation of Themes | 26 | Were themes identiﬁed in advance or derived from the data? | 13-14 |
| Software | 27 | What software, if applicable, was used to manage the data? | 14 |
| Participant Checking | 28 | Did participants provide feedback on the ﬁndings? | No |
| Reporting | | | |
| Quotations Presented | 29 | Were participant quotations presented to illustrate the themes/ﬁndings? Was each quotation identiﬁed? e.g., participant number | Table 3 |
| Data and ﬁndings Consistent | 30 | Was there consistency between the data presented and the ﬁndings? | Page 16, Table 3 & Pages 18-19, 21-24 |
| Clarity of Major Themes | 31 | Were major themes clearly presented in the ﬁndings? | 18 |
| Clarity of Minor Themes | 32 | Is there a description of diverse cases or discussion of minor themes? | Table 3 |

* Loction on original submission (page number will be different on published version)

Tong A, Sainsbury P, Craig J. Consolidated criteria for reporting qualitative research (COREQ): a 32-item checklist for interviews and focus groups. Int J Qual Health Care. 2007;19(6):349-357.

**Table S2:** STROBE Guideline - checklist of items that should be included in reports of observational studies

|  | **Item No** | **Recommendation** | **Located, Page** |  |
| --- | --- | --- | --- | --- |
| **Title and abstract** | 1 | (a) Indicate the study’s design with a commonly used term in the title or the abstract | 1 |  |
|  |  | (b) Provide in the abstract an informative and balanced summary of what was done and what was found | 1-2 |  |
| **Introduction** | | |  |  |
| Background/rationale | 2 | Explain the scientific background and rationale for the investigation being reported | 3-6 |  |
| Objectives | 3 | State specific objectives, including any prespecified hypotheses | 5-6 |  |
| Methods | | |  |  |
| Study design | 4 | Present key elements of study design early in the paper | 6 |  |
| Setting | 5 | Describe the setting, locations, and relevant dates, including periods of recruitment, exposure, follow-up, and data collection | 6-7 |  |
| Participants | 6 | (a) Cohort study—Give the eligibility criteria, and the sources and methods of selection of participants. Describe methods of follow-up  Case-control study—Give the eligibility criteria, and the sources and methods of case ascertainment and control selection. Give the rationale for the choice of cases and controls  Cross-sectional study—Give the eligibility criteria, and the sources and methods of selection of participants | 7 |  |
|  |  | (b) Cohort study—For matched studies, give matching criteria and number of exposed and unexposed  Case-control study—For matched studies, give matching criteria and the number of controls per case | NA |  |
| Variables | 7 | Clearly define all outcomes, exposures, predictors, potential confounders, and effect modifiers. Give diagnostic criteria, if applicable | 11-15 |  |
| Data sources/ measurement | 8 | For each variable of interest, give sources of data and details of methods of assessment (measurement). Describe comparability of assessment methods if there is more than one group | 11-15 |  |
| Bias | 9 | Describe any efforts to address potential sources of bias | 14 |  |
| Study size | 10 | Explain how the study size was arrived at | 6 |  |
| Quantitative variables | 11 | Explain how quantitative variables were handled in the analyses. If applicable, describe which groupings were chosen and why | 11-15 |  |
| Statistical methods | 12 | (a) Describe all statistical methods, including those used to control for confounding | 11-15 |  |
|  |  | (b) Describe any methods used to examine subgroups and interactions | 11-15 |  |
|  |  | (c) Explain how missing data were addressed | 13 |  |
|  |  | (d) Cohort study—If applicable, explain how loss to follow-up was addressed  Case-control study—If applicable, explain how matching of cases and controls was addressed  Cross-sectional study—If applicable, describe analytical methods taking account of sampling strategy | 11 |  |
|  |  | (e) Describe any sensitivity analyses | NA |  |
| **Results** | | |  | |
| Participants | 13* | (a) Report numbers of individuals at each stage of study—eg numbers potentially eligible, examined for eligibility, confirmed eligible, included in the study, completing follow-up, and analysed | 15 | |
|  |  | (b) Give reasons for non-participation at each stage | 15 | |
|  |  | (c) Consider use of a flow diagram |  | |
| Descriptive data | 14* | (a) Give characteristics of study participants (eg demographic, clinical, social) and information on exposures and potential confounders | 15 & Table 2 | |
|  |  | (b) Indicate number of participants with missing data for each variable of interest | Table 2 | |
|  |  | (c) Cohort study—Summarise follow-up time (eg, average and total amount) | 6 | |
| Outcome data | 15* | Cohort study—Report numbers of outcome events or summary measures over time | 15-21, Figure 2, & Figure 3 | |
|  |  | Case-control study—Report numbers in each exposure category, or summary measures of exposure |  | |
|  |  | Cross-sectional study—Report numbers of outcome events or summary measures |  | |
| Main results | 16 | (a) Give unadjusted estimates and, if applicable, confounder-adjusted estimates and their precision (eg, 95% confidence interval). Make clear which confounders were adjusted for and why they were included | 14 & 20 | |
|  |  | (b) Report category boundaries when continuous variables were categorized | Table S5 | |
|  |  | (c) If relevant, consider translating estimates of relative risk into absolute risk for a meaningful time period | NA | |
| Other analyses | 17 | Report other analyses done—eg analyses of subgroups and interactions, and sensitivity analyses | 20-21 and Figure 3 | |
| **Discussion** | | |  | |
| Key results | 18 | Summarise key results with reference to study objectives | 15-21 | |
| Limitations | 19 | Discuss limitations of the study, taking into account sources of potential bias or imprecision. Discuss both direction and magnitude of any potential bias | 24-24 | |
| Interpretation | 20 | Give a cautious overall interpretation of results considering objectives, limitations, multiplicity of analyses, results from similar studies, and other relevant evidence | 24-26 | |
| Generalisability | 21 | Discuss the generalisability (external validity) of the study results | 24-25 | |
| **Other information** | | |  | |
| Funding | 22 | Give the source of funding and the role of the funders for the present study and, if applicable, for the original study on which the present article is based | 27 | |

* Loction on original submission (page number will be different on published version)

von Elm E, Altman DG, Egger M, Pocock SJ, Gotzsche PC, Vandenbroucke JP. The Strengthening the Reporting of Observational Studies in Epidemiology (STROBE) Statement: guidelines for reporting observational studies.

**Table S3:** TIDieR Guideline – checklist of information to include when describing an intervention

| **Item number** | **Item number** | **Located, Page *** |
| --- | --- | --- |
|  | **BRIEF NAME** |  |
| **1.** | Provide the name or a phrase that describes the intervention. | 2 |
|  | **WHY** |  |
| **2.** | Describe any rationale, theory, or goal of the elements essential to the intervention. | 9-13 |
|  | **WHAT** |  |
| **3.** | Materials: Describe any physical or informational materials used in the intervention, including those provided to participants or used in intervention delivery or in training of intervention providers. Provide information on where the materials can be accessed (e.g. online appendix, URL). | 9-13 and supplementary material |
| **4.** | Procedures: Describe each of the procedures, activities, and/or processes used in the intervention, including any enabling or support activities. | 9-13 |
|  | **WHO PROVIDED** |  |
| **5.** | For each category of intervention provider (e.g. psychologist, nursing assistant), describe their expertise, background and any specific training given. | 12 |
|  | **HOW** |  |
| **6.** | Describe the modes of delivery (e.g. face-to-face or by some other mechanism, such as internet or telephone) of the intervention and whether it was provided individually or in a group. | 9-13 |
|  | **WHERE** |  |
| **7.** | Describe the type(s) of location(s) where the intervention occurred, including any necessary infrastructure or relevant features. | 9-13 |
|  | **WHEN and HOW MUCH** |  |
| **8.** | Describe the number of times the intervention was delivered and over what period of time including the number of sessions, their schedule, and their duration, intensity or dose. | 9-13 |
|  | **TAILORING** |  |
| **9.** | If the intervention was planned to be personalised, titrated or adapted, then describe what, why, when, and how. | NA |
|  | **MODIFICATIONS** |  |
| **10.^ǂ^** | If the intervention was modified during the course of the study, describe the changes (what, why, when, and how). | NA |
|  | **HOW WELL** |  |
| **11.** | Planned: If intervention adherence or fidelity was assessed, describe how and by whom, and if any strategies were used to maintain or improve fidelity, describe them. | NA |
| **12.^ǂ^** | Actual: If intervention adherence or fidelity was assessed, describe the extent to which the intervention was delivered as planned. | NA |

* Loction on original submission (page number will be different on published version)

**Table S4:** Text messages mapped to behaviour change techniques as per the behaviour change technique taxonomy^1^ and mechanism of action^2^

| **Behaviour change technique** | **Text messages mapped to the BCT**  **Frequency (%)** |
| --- | --- |
| Problem solving (includes relapse prevention) | 20 (24%) |
| Instruction on how to perform the behaviour | 14 (17%) |
| Information about health consequence | 10 (12%) |
| Behaviour substitution | 10 (12%) |
| Goal setting (behaviour) | 8 (10%) |
| Social support (unspecified) | 7 (8%) |
| Self-monitoring of behaviour | 4 (5%) |
| Habit formation | 4 (5%) |
| Social support (practical) | 3 (4%) |
| Self-reward | 3 (4%) |
| Self-monitoring of outcome(s) of behaviour | 1 (1%) |

^1^ Michie, S., Richardson, M., Johnston, M., Abraham, C., Francis, J., Hardeman, W., ... & Wood, C. E. (2013). The behaviour change technique taxonomy (v1) of 93 hierarchically clustered techniques: building an international consensus for the reporting of behaviour change interventions. Annals of behavioural medicine, 46(1), 81-95.

^2^ Johnston, M., Carey, R. N., Connell Bohlen, L. E., Johnston, D. W., Rothman, A. J., de Bruin, M., ... & Michie, S. (2021). Development of an online tool for linking behaviour change techniques and mechanisms of action based on triangulation of findings from literature synthesis and expert consensus. Translational behavioural medicine, 11(5), 1049-1065.

n(%); frequency and percentage

**Table S5:** Outline of the digital online resources and videos

| **Diet upgrades after surgery:**  “After surgery, you will upgrade the texture of your diet at regular intervals, progressing from fluids to puree, to soft foods before being able to include a variety of textures into a balanced diet. Videos and information sheets have been created to support you through these upgrades.” |
| --- |
| *Post-surgery diet overview and Stage 1 – Fluids*   - Video: 4 minutes 25 seconds in duration - Handout: “Stage 1: Fluids (liquids) diet after bariatric surgery” |
| *Stage 2 – Puree*   - Video: 6 minutes 31 seconds in duration - Handout: “Stage 2: Puree diet upgrade after bariatric surgery” |
| *Stage 3 – Soft*   - Video: 3 minutes 56 seconds in duration - Handout: Stage 3: “Soft diet upgrade after bariatric surgery” |
| *Stage 4 – Healthy eating after surgery*   - Video: 4 minutes 40 seconds in duration - Handout: “Stage 4: Solid (normal texture) foods – Healthy eating after bariatric surgery” |
| **Annual Review Checklist**:  “After surgery, it is important that your GP is monitoring your post- surgical care yearly, or more often if required. This check list provides a guide for lifelong post-bariatric surgery management.”   - Handout: “Annual Review Checklist” |
| **Medication changes after surgery**:  “After bariatric surgery your medications may be absorbed differently due to changes in the size and shape of your digestive system. This handout may provide you with information on managing your medicines after surgery.   - Handout: “Changes to your medicines after bariatric surgery for patients who have had a sleeve gastrectomy, Roux-en-Y bypass (RYGB) or other sleeve/bypass procedures.” |
| **Healthy lifestyle support:**  “These sites provide information on healthy eating and exercise post-surgery. To help achieve your health- related goals and maintain long-term weight loss, it’s important to follow a healthy diet and include regular exercise”   - Link to external websites: “Health at home”, “Get healthy program – information and coaching”, and “healthy weight guide”. |
| **Post operative concerns:**  “These videos will provide you with information on concerns patients commonly report after surgery, some tips on how to manage these symptoms and guidelines for when you should contact the team.” |
| *Commonly reported concerns:*   - Video: 3 minutes 20 seconds in duration |
| *Post-surgical concerns:*   - Video: 5 minutes 19 seconds in duration |
| **Coping Tools:**  “This section provides information on tools you may find useful to help cope when preparing for surgery and adjusting post- surgery.” |
| *Goal setting*   - Video: 3 minutes and 7 seconds in duration - Handouts: “My action plan”, “How to set goals”, “Goal setting plan” - Link to external websites: “Stage of change”, “Setting goals”, and “Defining goals” |
| *Mindful eating*   - Video: 2 minutes and 7 seconds in duration - Handout: “Mindfulness in everyday life” - Link to external website: “Moving & Eating Mindfully” |

**Table S6:** Survey assessing the acceptability and usability of the mHealth program (N=16)

| **The 19-item survey questions** | **Frequency** | **Percentage^a^** |
| --- | --- | --- |
| **Modality** |  |  |
| *Q1: During the last 12 months I received (n=16)* |  |  |
| Text messages only | 8 | 50.0% |
| Email newsletter only | 1 | 6.0% |
| Both text messages and email newsletter | 7 | 44.0% |
| *Q2: During the last 12 months (n=16)* |  |  |
| I visited the hospital’s bariatric surgery website | 4 | 25.0% |
| I watched a video on the hospital’s bariatric surgery website | 2 | 12.5% |
| Did not visit the Hospital bariatric surgery website or watch any of the videos | 10 | 62.5% |
| **Specific to receiving text messages (n=15)** |  |  |
| *Q3: The text messages I received were too regular/frequent* |  |  |
| No | 14 | 93.0% |
| Yes | 1 | 7.0% |
| *Q4: When receiving a text message, I would* |  |  |
| Always read it | 14 | 93.3% |
| Read it most of the time | 1 | 6.7% |
| Read it half of the time | 0 | 0.0% |
| Read it some of the time | 0 | 0.0% |
| Never read it | 0 | 0.0% |
| *Q5: The text messages I received were easy to understand* |  |  |
| Strongly agree | 9 | 60.0% |
| Agree | 6 | 40.0% |
| Neither agree or disagree | 0 | 0.0% |
| Disagree | 0 | 0.0% |
| Strongly disagree | 0 | 0.0% |
| *Q6: The text messages I received were useful in supporting new behaviours* |  |  |
| *Strongly agree* | 5 | 33.3% |
| Agree | 8 | 53.3% |
| Neither agree or disagree | 2 | 13.3% |
| Disagree | 0 | 0.0% |
| Strongly disagree | 0 | 0.0% |
| *Q7: The text messages I received motivated me to set goals or keep track of my food or exercise* |  |  |
| Strongly agree | 3 | 20.0% |
| Agree | 5 | 33.3% |
| Neither agree or disagree | 6 | 40.0% |
| Disagree | 1 | 6.7% |
| Strongly disagree | 0 | 0.0% |
| *Q8: The text messages I received motivated me to change my diet* |  |  |
| Strongly agree | 2 | 13.3% |
| Agree | 4 | 26.7% |
| Neither agree or disagree | 7 | 46.7% |
| Disagree | 2 | 13.3% |
| Strongly disagree | 0 | 0.0% |
| *Q9: The text messages I received made me exercise more* |  |  |
| Strongly agree | 2 | 13.3% |
| Agree | 3 | 20.0% |
| Neither agree or disagree | 7 | 46.7% |
| Disagree | 2 | 13.3% |
| Strongly disagree | 0 | 0.0% |
| Did not wish to disclose | 1 | 6.7% |
| **Specific to receiving email newsletter (n=8)** |  |  |
| *Q10: The email newsletter had useful information in it* |  |  |
| Yes | 7 | 87.5% |
| No | 1 | 12.5% |
| *Q11: The email newsletter I received was too regular/frequent* |  |  |
| Yes | 1 | 12.5% |
| No | 7 | 87.5% |
| *Q12: When receiving an email newsletter, I would* |  |  |
| Always read it | 6 | 75.0% |
| Read it most of the time | 1 | 12.5% |
| Read it half of the time | 1 | 12.5% |
| Read it some of the time | 0 | 0.0% |
| Never read it | 0 | 0.0% |
| *Q13: The email newsletter I received was easy to understand* |  |  |
| Strongly agree | 4 | 50.0% |
| Agree | 3 | 37.5% |
| Neither agree or disagree | 1 | 12.5% |
| Disagree | 0 | 0.0% |
| Strongly disagree | 0 | 0.0% |
| **Specific to accessing the hospital’s website (n=4)** |  |  |
| *Q14: The information on the hospital’s bariatric surgery website was useful* |  |  |
| Yes | 3 | 75.0% |
| No | 0 | 0.0% |
| Did not wish to disclose | 1 | 25.0% |
| *Q15: The information on the hospital’s bariatric surgery website was easy to understand* |  |  |
| Yes | 3 | 75.0% |
| No | 0 | 0.0% |
| Did not wish to disclose | 1 | 25.0% |
| *Q16: On the Hospital bariatric surgery website I preferred* |  |  |
| Watching videos | 2 | 50.0% |
| Reading information | 0 | 0.0% |
| Both watching videos and reading information | 0 | 0.0% |
| Did not wish to disclose | 2 | 50.0% |
| **Specific to accessing the hospital’s videos (n=2)** |  |  |
| *Q17: The videos on the hospital’s bariatric surgery website were useful* |  |  |
| Yes | 2 | 100.0% |
| No | 0 | 0.0% |
| *Q18: The videos on the Hospital bariatric surgery website were easy to understand* |  |  |
| Yes | 2 | 100.0% |
| No | 0 | 0.0% |
| **Additional qualitative questions (n=16)** |  |  |
| *Q19: Do you have any suggestions for improvements or additional comments you would like to make?* | 2 | 12.5% |

^a^ Total percentage, with all percentages referring to the valid data available for the variable

**Table S7:** Semi-structured interview guide

| **Focus point** | **Key questions and prompts** |
| --- | --- |
| 1. Warm up, rapport building, motives | - Can you tell me a little bit about why you signed up to receive the text messages and email newsletter? - Did you opt out of any of the services and why yes/no? - Can you tell me if the text messages and email newsletter were what you expected they would be? |
| 1. Program delivery methods | - Was there a particular delivery method that you found more helpful, out of the text messages, emails, website and videos on the website. - Our system can send a text any time of day. were you comfortable at receiving them at the set time of 8:00 am? *(Added after the pilot interviews)** |
| 1. Text message element | - Can you give me an example of a text message you liked the most / and or the least? - Is there a particular text that sticks out in your mind? - We had four or five different styles of text messages, some that would give you information, some a reminder on how to do something, some to set goals; do you remember those type of questions, which ones did you like the most? *(Added after the pilot interviews)** |
| 1. Email newsletter element | - Patients that we spoke to in the past mentioned they would like an email newsletter. Since you have been receiving the email newsletter, what are your thoughts on delivering extra information to patients via email? - Do you recall clicking on any of the links within the email newsletter? What made you click? - Can you give me an example of a topic in the email newsletter you liked the most / and or the least? |
| 1. Online resources and videos element | - Have you utilised any information or resources on the Bariatric Surgery Services website? - If no: are they aware of the website. - If yes: what have you found the most helpful? |
| 1. Alternate elements | - Are there other options of extra add-ons that the hospital could offer patients via technology that might be more attractive to you? *(Added after the pilot interviews)** |
| 1. Behaviour change | - When suggestions presented in a text message; how likely were you to do what it suggested? - Can you recall what made it hard to put suggestions into place? - Was there anything that you were prompted by the text-messages to started doing that was new to you? Why did you make this change? Do you think you would have done this without the text message? |
| 1. Goal setting | - What has been your experience with setting goals for yourself over the past few months? - Were your goals related to information from text messages, the newsletter, or other source? - Are you able to tell me about one of the goals? |
| 1. Self-monitoring | - What impact do you think the text messages had on tracking and keeping record of your progress, exercise, or food? - Did you start self-monitoring because of the text messages or do this before it? |
| 1. Maintaining behaviours and strategies | - How confident are you to set your own goals, track your progress, and keep up new habits moving forward? - Do you foresee any challenges moving forward? *(Added after the pilot interviews)** |
| 1. Program content | - Are there any topics or information that you would have liked to have seen in the text messages or email newsletter that we missed? Or any suggestions for improvements? - How can we benefit more people? |
| 1. Usual care shifted to telehealth due to COVID-19 | - Over the past few months, what has been your experience with ‘telehealth’ (having your appointments over the phone or via video call)? - What are your thoughts on this and is this an option you would want to continue to be available as an alternative to coming in for in person appointment? |
| 1. Closing | - Is there anything that you think would be important to mention that we haven’t covered? |

***** The first three interviews conducted served as a pilot test of the interview guide; these interviews were included in the final analysis. Two research team members ([blinded]) reviewed the interview guide after the pilot interviews to ensure relevant responses were drawn. After which, four additional questions were added to the interview guide.

**Table S8**: Baseline demographics between participants recruited into the intervention at 12-months, 3-months, or 6-months

| **Characteristic** | **Recruited into intervention at 3-months (n=16)**  **Frequency (%)** | **Recruited into intervention at 6-months (n=9)**  **Frequency (%)** | **Recruited into intervention at 12-months (n=22)**  **Frequency (%)** |
| --- | --- | --- | --- |
| Sex, Female | 12 (75.0%) | 6 (66.7%) | 13 (59.1%) |
| Age (years) ^a^ | 52.1 ± 10.0 | 52.6 ± 7.0 | 57.0 ± 9.9 |
| Type of surgery |  |  |  |
| Roux-en-Y gastric bypass | 14 (87.5%) | 8 (88.9%) | 20 (90.9%) |
| Laparoscopic sleeve gastrectomy | 1 (6.3%) | 1 (11.1%) | 2 (9.1%) |
| Mini bypass | 1 (6.3%) | 0 (0.0%) | 0 (0.0%) |
| Single loop | 0 (0.0%) | 0 (0.0%) | 0 (0.0%) |
| Pre-operative anthropometry |  |  |  |
| Height (m) ^a^ | 1.7 ± 0.1 | 1.7 ± 0.1 | 1.7 ± 0.1 |
| Weight (kg) ^a^ | 121.9 ± 18.8 | 113. 2 ± 16.1 | 119.0 ± 14.7 |
| Body mass index (kg/m^2^) ^a^ | 43.9 ± 6.4 | 40.8 ± 3.7 | 41.1 ± 6.2 |
| Excess weight (kg) ^a^ | 57.9 ± 12.3 | 51.5 ± 10.0 | 55.3 ± 14.0 |
| Employment status |  |  |  |
| Full-time | 6 (37.5%) | 4 (44.4%) | 10 (45.5%) |
| Part-time | 3 (18.8%) | 3 (33.3%) | 4 (18.2%) |
| Home duties/retired | 7 (43.8%) | 2 (22.2%) | 8 (36.4%) |
| Unemployed | 0 (0.0%) | 0 (0.0%) | 0 (0.0%) |
| Aboriginal and Torres Strait Islander |  |  |  |
| Does not identify | 11 (68.8%) | 6 (66.7%) | 14 (63.6%) |
| Aboriginal | 3 (18.8%) | 1 (1.11%) | 4 (18.2%) |
| Torres Strait Islander | 2 (12.5) | 0 (0.0%) | 0 (0.0%) |
| Aboriginal and Torres Strait Islander | 0 (0.0%) | 2 (22.2%) | 1 (4.5%) |
| Did not wish to disclose | 0 (0.0%) | 0 (0.0%) | 3 (13.6%) |
| Length of hospital stay (days) ^b^ | 2 (1-6) | 2 (1-7) | 2 (1-7) |

^a^ mean ± standard deviation; ^b^ median (range)
